# Introduction and Rollout of a New Group A Meningococcal Conjugate Vaccine (PsA-TT) in African Meningitis Belt Countries, 2010–2014

**DOI:** 10.1093/cid/civ551

**Published:** 2015-11-09

**Authors:** Mamoudou H. Djingarey, Fabien V. K. Diomandé, Rodrigue Barry, Denis Kandolo, Florence Shirehwa, Clement Lingani, Ryan T. Novak, Carol Tevi-Benissan, William Perea, Marie-Pierre Preziosi, F. Marc LaForce

**Affiliations:** 1 Inter-country Support Team for West Africa, World Health Organization, Ouagadougou, Burkina Faso; 2 Centers for Disease Control and Prevention, Atlanta, Georgia; 3 World Health Organization, Nigeria Country Office, Abuja, Nigeria; 4 World Health Organization, Regional Office for Africa, Brazzaville, Republic of Congo; 5 Department of Pandemic and Epidemic Diseases; 6 Department of Immunization, Vaccines and Biologicals, World Health Organization, Geneva, Switzerland; 7 Department of Meningitis Vaccine Project, PATH, Ferney-Voltaire, France; 8 Department of Serum Institute of India, Ltd, Pune

**Keywords:** meningococcal group A, meningitis belt, Africa, PsA-TT, rollout plan

## Abstract

***Background.*** A group A meningococcal conjugate vaccine (PsA-TT) was developed specifically for the African “meningitis belt” and was prequalified by the World Health Organization (WHO) in June 2010. The vaccine was first used widely in Burkina Faso, Mali, and Niger in December 2010 with great success. The remaining 23 meningitis belt countries wished to use this new vaccine.

***Methods.*** With the help of African countries, WHO developed a prioritization scheme and used or adapted existing immunization guidelines to mount PsA-TT vaccination campaigns. Vaccine requirements were harmonized with the Serum Institute of India, Ltd.

***Results.*** Burkina Faso was the first country to fully immunize its 1- to 29-year-old population in December 2010. Over the next 4 years, vaccine coverage was extended to 217 million Africans living in 15 meningitis belt countries.

***Conclusions.*** The new group A meningococcal conjugate vaccine was well received, with country coverage rates ranging from 85% to 95%. The rollout proceeded smoothly because countries at highest risk were immunized first while attention was paid to geographic contiguity to maximize herd protection. Community participation was exemplary.

A new group A meningococcal conjugate vaccine (PsA-TT) was developed by the Meningitis Vaccine Project (MVP) and manufactured at the Serum Institute of India, Ltd (SIIL) [1]. The vaccine was prequalified by the World Health Organization (WHO) in June 2010. PsA-TT clinical trials that had been done in persons aged 1–29 years in India and in Africa showed that the vaccine had a safety profile similar to that of licensed meningococcal polysaccharide vaccines and produced stronger and more persistent immunological response against group A *Neisseria meningitidis* [2]. Initial evaluation of the vaccine's effectiveness and its safety profile during first introduction showed that the vaccine is safe, significantly reduced meningococcal carriage, and dramatically reduced disease incidence [3–5].

The success of PsA-TT during first introduction in Burkina Faso, Mali, and Niger generated great interest in the vaccine in other “meningitis belt” countries. This article describes the steps that were taken to ensure that the rollout of the PsA-TT vaccine in the meningitis belt countries occurred smoothly.

## VACCINE ROLLOUT PLAN AND RISK TOOL

The PsA-TT introduction strategy targeted 1- to 29-year-olds through mass vaccination campaigns. The specific objectives of the program were to vaccinate at least 90% of the target population, to monitor vaccine adverse events following immunization (AEFIs), and to assess the impact of vaccine introduction on group A meningococcal carriage and disease incidence while monitoring meningitis outbreaks due to other strains.

Despite many efforts to develop predictive models, it has not been possible to predict where and when meningococcal epidemics will take place. Nonetheless, decisions as to where and when to introduce the PsA-TT vaccine had to be made to prioritize countries so that plans could be developed. A methodology was developed for sequencing country introduction that was based on disease incidence and burden and the ability of countries to mount vaccination campaigns [6]. Using this tool, countries were then ranked into 5 categories:

- Group 1: Countries with high epidemic risk and high disease burden (Burkina Faso, Chad, Ethiopia, Mali, Niger, Nigeria, and Sudan);
- Group 2: Countries with high epidemic risk, but low disease burden (Benin, Cameroon, and Ghana);
- Group 3: Countries with low epidemic risk, but high disease burden (Democratic Republic of Congo, South Sudan);
- Group 4: Countries with an intermediate epidemic risk (>4 small-scale epidemics (<3000 cases) since 1970 (Côte d'Ivoire, Guinea, Senegal, Togo, Uganda);
- Group 5: Countries with low epidemic risk and low disease burden (Burundi, Central African Republic, Eritrea, The Gambia, Guinea-Bissau, Kenya, Mauritania, Rwanda, and Tanzania).

At a more granular level, subnational district data were also used to more precisely define high-risk areas that could be targeted earlier. Thus, the prioritization tool allowed for the creation of a phased vaccine introduction plan that was sensitive to the level of the risk of transmission in specific areas within a country. For example, northern Ghana and northern Nigeria, with high disease incidence and disease burden, were considered to be at much higher risk of meningitis epidemics than were southern Ghana and southern Nigeria. Although Nigeria was ranked in group 1, given its size and population, vaccine introduction in this country spanned 4 years. A country's participation in the PsA-TT clinical trials was also considered a positive criterion that favored early introduction.

**Figure 1.**
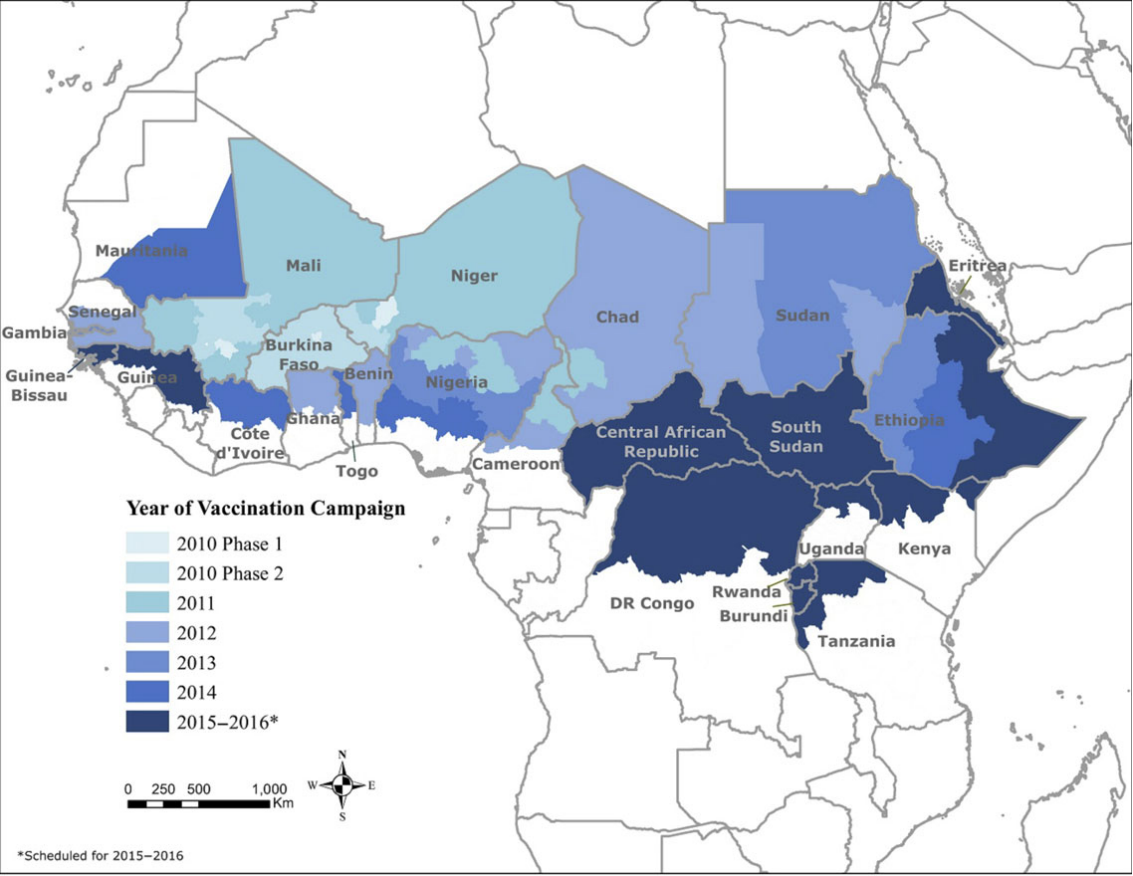
Group A meningococcal conjugate vaccine (PsA-TT) rollout (2010–2014) in countries of the African meningitis belt. Source: World Health Organization Inter-country Support Team report on PsA-TT campaigns (meningvax.org).

Last, the quality of the country's preparedness and its commitment to support 50% of the operational cost of the mass campaign were also used as criteria to select countries. The vaccine manufacturer (SIIL) also indicated that if vaccine orders were to be >40 million doses, the orders would have to be placed before 15 April of each year to ensure that there was sufficient production time. Based on all these criteria, a country-based introduction scheme was established (Figure 1).

## PREIMPLEMENTATION ACTIVITIES

### Resource Mobilization

The cost per vaccinated individual was estimated at US$1.40, based on historical data and the cost estimates from the first introductions in 2010 in Burkina Faso, Mali, and Niger. The cost figure included the vaccine, needles, and syringes as well as operational and infrastructure costs. The total cost to implement PsA-TT introduction from 2011 to 2014 was estimated at US$253 million and included US$63.8 million of in-country financial commitment to support operational costs of vaccination campaigns.

The initial 2010 introduction of PsA-TT in Burkina Faso, Mali, and Niger was endorsed by Gavi, which approved US$29.5 million in direct support. As country introductions ensued, each country developed its own fundraising strategy that combined Gavi support, in-country funds for operational costs, and other resources from bilateral donors. Beginning in 2011, countries wishing to conduct PsA-TT campaigns were required to submit their proposals to the Gavi secretariat through a new application process that included the prioritization exercise. At the country level, the campaign budgets were developed, endorsed, and modified, particularly as regional needs became more obvious.

### Coordination

International coordination was established through regular teleconferences (TCs) led by the MVP team at the WHO Inter-country Support Team for West Africa in Ouagadougou, Burkina Faso, and the WHO Regional Office for Africa. The TCs involved implementing countries as well as all the partners (WHO, United Nations Children's Fund [UNICEF], Centers for Disease Control and Prevention [CDC], Médecins Sans Frontières [MSF], Bill & Melinda Gates Foundation, SIIL, and Gavi) that were supporting the countries. The TCs were very important in monitoring progress, identifying constraints, and sharing experiences.

At the country level, coordination groups were created at national, regional/state, and district levels. Working groups for training, logistics, monitoring, and evaluation and social mobilization were established, and each focused on its specific area. A country-specific chronogram was developed and endorsed by the core group to guide the team while ensuring that activities unfolded in a timely manner. Membership of the working groups included ministry of health (chairmanship), key ministries of health departments (Expanded Programme on Immunization [EPI], surveillance, vaccine regulatory), and partners such as WHO, UNICEF, CDC, and MSF.

Consultative stakeholders’ meetings were held with health authorities from the implementing regions/states/districts to share the rationale for the campaign lessons learned from previous phases of the campaign, and to discuss responsibilities regarding budget, logistics, and community sensitization and mobilization.

### Activities Conducted by Working Groups

The working groups met regularly to review activities.

#### Operation Team

The operation team reviewed the field guide, data tools, operational guidelines, and daily implementation plans to incorporate lessons learned from previous campaigns. It had the overall responsibility for planning and ensuring the implementation of activities.

#### Microplanning

A microplan template that addressed campaign logistics and communication strategies aimed at target age groups was reviewed. The national level planned and conducted a training of trainers on microplanning for the regions/states/districts, and cascaded training was done to the district level. Training at the national level included district, regional, and state health officials; epidemiologists; health educators; cold chain officers; and partners. Training material included the rationale for the PsA-TT campaign, the overall strategy, microplanning, plan for logistics, AEFI monitoring, surveillance, and waste management. The same content was maintained at all levels. Microplanning was extended to the subdistrict level.

The planning process included meetings with traditional leaders and other influential persons. A template that captured all requirements was used by each subdistrict unit (ward). Planning benefited greatly from the geographic information system (GIS) maps and previous immunization activities. Target population data were generated at the ward level, and this information was used to determine the number of teams needed in a ward. Other parameters included distances between settlements and the terrain between districts. The GIS maps were used in some regions/states such as Kano and Sokoto in Nigeria to identify vaccination sites and the size of mobile teams. The microplans enumerated all special places where target populations could be found such as schools, mosques, churches, bus terminals, and markets. Some areas were initially left out but were later identified during the campaigns such as workplaces, hotels, and restaurants. These were important areas because they employed many males aged 16–29 years. National microplanning verification meetings for regions/states were conducted to establish adequacy of the plans in light of the resources that were assigned.

#### Logistics

Logistic working groups developed a logistic plan, conducted cold chain assessments, developed a waste management plan, repaired gaps in cold chains where needed, developed tools to help countries to establish cold chain adequacy, developed vaccine and material distribution plans, and identified holding points for vaccines. When cold chain gaps were identified, they assisted the procurement department in identifying appropriate incinerators and installation sites for the incinerators. All 0.5-mL Auto-Disable (AD) syringes, 5-mL AD syringes, safety boxes, and vaccines arrived on time. Vaccines and other supplies were distributed to regions, states, and districts according to country logistic plans previously validated at the national level.

#### Social Mobilization

Social mobilization activities were conducted at national, regional, state, and district levels. The social mobilization working groups held meetings with regions, states, and districts to develop plans and budgets. They explored information management with health, education, and information authorities; conducted sensitization meetings with media houses; developed materials and radio jingles; created messages for specific age groups; and printed banners and posters in official and local languages. In all regions, states, and districts, social mobilization activities that included radio jingles, radio discussions, meetings with traditional leaders, and key stakeholders were conducted. Ministries of information and education were part of the social mobilization working group at regional, state, and district levels. At the ward level, planning meetings were used to create awareness. A town crier was identified for each team. A month before the campaign, a crisis communication workshop was organized with key officials and representatives from ministries of health, to brief them on how to deal with situations that might jeopardize the campaign or be detrimental to the country's immunization program. These included critical AEFIs such as death, adverse rumors, and refusals.

#### Training

Training was conducted at all levels. Presentations and agendas were developed at the national level to ensure standardization. Pre- and posttests were administered. Training evaluations were carried out during each training session and immediate feedback was given to facilitators. This helped in improving the quality of the presentations. Training at regional, state, and district levels were conducted within 14 days of the start of the campaign.

#### Management of AEFIs

Each country received assistance in setting up its AEFI expert committee for causality assessment. A review of the AEFI guidelines was conducted. The guidelines were printed and distributed to all regions, states, and districts. All regional, state, and district teams were trained on AEFI management. AEFI kits were provided at vaccination posts. The kits contained adrenaline, paracetamol, syringes, and chlorpheniramine. At referral health centers, adrenaline, cortisone, saline drip, infusion sets, chlorpheniramine, and syringes were available in the event of serious allergic and anaphylactic reactions. Data capturing tools were printed at the central level and distributed. Each post had reporting and line listing forms, and the district level had the investigation, laboratory, and summary forms for AEFIs.

#### Strengthening Capacities for Meningitis Case-Based Surveillance

Additional efforts were made to strengthen capacity for case-based meningitis surveillance prior to the introduction of the PsA-TT vaccine as a platform to measure vaccine effectiveness and impact. Specific activities included the revisions of guidelines and tools, laboratory capacity building, improvement of data management, and training of surveillance officers.

## VACCINATION CAMPAIGNS

Mass vaccination campaigns with PsA-TT that targeted 1- to 29-year-olds were done. Independent monitoring was conducted during the campaign (in-process monitoring) and during the first week after the completion of the campaign (end-process monitoring) by independent monitors using standardized forms. They conducted household visits by randomly selecting 2 settlements in a catchment area and visited 5 households in each settlement. In each household visited, information was systematically collected on the following: (1) the number of people physically present in the household by age group; (2) the total number of persons with a vaccination card; (3) the total number of persons not vaccinated; (4) the reason why any individual was not vaccinated (absence from the area, active decision not to be vaccinated, vaccination post too far away, too long a wait for vaccination, no vaccinator at the vaccination post, lack of information about the vaccine); (5) the sources of information about the vaccination; and (6) the awareness of potential side effects from the vaccine.

In addition to the household visits, a group of 20 individuals aged 1–29 years was selected randomly in the streets, and similar information about vaccination was collected. Confirmation of receipt of vaccine relied on a vaccination card that indicated that PsA-TT had been given. Vaccination coverage was calculated by dividing the number of people with vaccination cards (numerator) by the total number of eligible people physically seen (denominator). Random coverage surveys were also conducted at least 1 month after the end of the vaccination campaigns. Two methods were used for conducting these surveys: the WHO EPI stratified cluster survey method [7], and the lot quality assurance sampling method [8].

## VACCINE ROLLOUT

**Figure 2.**
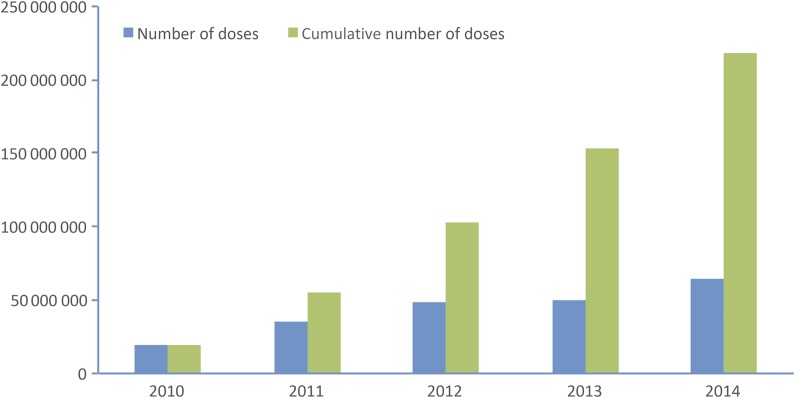
Annual and cumulative doses used in the group A meningococcal conjugate vaccine (PsA-TT) rollout from 2010 to 2014. Source: WHO Inter-country Support Team report on PsA-TT campaigns (meningvax.org).

From 2010 to 2014, PsA-TT was introduced in 15 countries of the African meningitis belt. The annual amounts of vaccine doses that were used ranged from about 20 million doses in the 2010 campaigns to >64 million doses during the 2014 campaigns, representing a cumulative total of 217 million Africans who were vaccinated over the ensuing 5 years (Figure 2). Most of the country vaccinations were done in the fourth quarter prior to the onset of the meningitis season in the first quarter of the following year. Table 1 summarizes the dates of introduction for individual countries, the at-risk populations, and the doses administered. Except for Guinea, all countries that fell into the WHO risk groups 1, 2, and 3 were immunized from 2010 to 2014. Guinea was scheduled to be immunized in 2014, but the campaign was canceled because of an Ebola outbreak. The 1- to 29-year-old populations in Mali, Burkina Faso, Niger, Chad, The Gambia, Sudan, and Ethiopia (3rd and final phased campaign to be conducted in 2016) were all vaccinated because the entire country was deemed high risk. Regional campaigns to cover high-risk areas were done in Nigeria, Cameroon, Ghana, Benin, Senegal, Côte d'Ivoire, Mauritania, and Togo. Figure 1 documents the vaccine rollout geographically. Whenever possible, contiguous areas were immunized from one year to the next. The 11 remaining countries (Central African Republic, Eritrea, Kenya, Burundi, Guinea, Guinea-Bissau, Rwanda, Uganda, Democratic Republic of Congo, South Sudan, and Tanzania) are scheduled for 2015–2016.

**Table 1.** Results From PsA-TT Vaccination Campaigns in 1- to 29-Year-Olds in 15 African Countries, 2010–2014

Countries	Regions, States, Districts Immunized	Year(s)	Target Population (1–29 y), No.	No. Immunized	Administrative Coverage, %	Random Surveys, %
Burkina Faso	Countrywide	2010	11 133 831	11 421 502	103	95.9 (95.0–96.7)^a^
Mali	Countrywide	2010–2012	10 854 599	11 109 484	102	96.0
Niger	Countrywide	2010–2012	10 870 817	10 575 365	97	91.0
Chad	Countrywide	2011–2012	9 223 913	8 732 251	95	Not conducted
Cameroon	4 northern regions	2011–2012, 2014	6 125 854	6 510 729	106	73.5
Nigeria	17 northern states	2011–2014	83 928 121	80 742 948	92	69.9
Benin	5 northern regions	2012	2 595 660	2 718 459	105	96.0
Ghana	3 northern regions	2012	3 098 348	3 038 393	98	90.1
Senegal	8 northern regions	2012, 2014	4 383 255	4 216 691	96	96.2
Sudan	Countrywide	2012, 2014	24 761 871	23 670 245	96	94.0
The Gambia	Countrywide	2013	1 177 923	1 228 419	104	96.6
Ethiopia	7 northern regions, 45 western districts	2013–2014	45 924 802	44 894 079	98	Not conducted
Mauritania	33 southern districts	2014	1 610 020	1 561 720	97	Not conducted
Togo	42 northern districts	2014	2 753 823	2 764 839	100.40	Not conducted
Côte d'Ivoire	25 northern districts	2014	4 271 001	4 587 056	107.40	Not conducted
Total	15 countries	2010–2014	222 713 838	217 772 180	97.8	

Source: World Health Organization Inter-country Support Team report on PsA-TT campaigns (meningvax.org).

^a^ Regional and weighted national PsA-TT vaccine coverage (95% confidence interval) [9].

The vaccine was well received by communities and authorities wherever it was introduced. The aggregate administrative coverage in the 15 countries was almost 98%. Administrative estimates of coverage always overestimate coverage because of the assumption that all vaccine doses were given to the correct age group. More rigorous coverage surveys were conducted during the first 3 years of introduction and for the most part confirmed that coverage in the correct age group was >90% (Table 1). A randomized stratified cluster survey was conducted in Burkina Faso in 2011 after the 2010 countrywide mass vaccination campaign [9]. The overall national coverage was 95.9% (range, 90.8%–98.3%) within the regions, and coverage was 97.7% in those aged 2–5 years, 97.4% in those aged 6–15 years, and 93.4% in those aged 16–30 years [9]. In Niger, vaccination coverage was estimated using a cluster-sampling survey and nested lot quality assurance analysis in the clustered samples to identify subpopulations with inacceptable vaccination coverage [10]. The overall coverage was estimated at 78% as documented by vaccination cards and 85% when verbal history of vaccination was included. Information on PsA-TT coverage was collected during cross-sectional carriage survey in Burkina Faso. Vaccination coverage measured varied by district, and was 93.6% in Bogodogo, 83.8% in Dandé, and 91.8% in Kaya [3]. When discrepancies were noted that suggested that less than adequate coverage had been reached (defined as <70%), “mop-up” campaigns were done in certain districts, as was the case in Cameroon and Nigeria.

**Figure 3.**
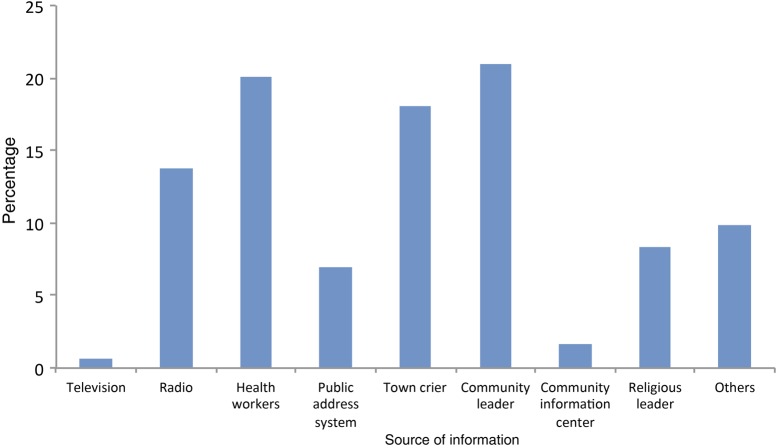
Percentage of source of information about the campaign among persons interviewed after the group A meningococcal conjugate vaccine (PsA-TT) 2012 campaign in Ghana (structured interview during cluster sample survey). Other sources of information included friends, school, at the borehole, neighbor, community surveillance volunteer, and marketplace. Source: WHO Inter-country support team report (2012).

The observed high performance of the vaccination program was achieved because of the quality of the district microplanning that was done. In addition, there was an overall soundness in the coordination of advocacy and social mobilization activities that used many channels to reach the target populations where they lived. Figure 3 summarizes data from a study in rural northern Ghana that explored how populations learned about the PsA-TT vaccination campaign. Results of the study indicated that multiple sources of information were required to reach these groups.

The monitoring of vaccine adverse events during the 10 days of mass vaccination campaigns and over the next 42 days following the end of the mass campaign did not reveal any safety issues [11]. The only negative vaccine-related event occurred in Gouro, Chad, in December 2013, where a small group of vaccinees developed a range of unusual symptoms after vaccination that were later identified as an instance of mass hysteria, an unusual phenomenon that has been well described in the context of mass immunization campaigns [12–14]. The episode is described in more detail in an accompanying communication article [15]. Initially, a Chadian reporter described paralysis in PsA-TT vaccine recipients. Careful clinical examinations by physicians and a neurologist revealed that there were no cases of paralysis and that all affected individuals fully recovered over the ensuing days.

## PSA-TT USE IN A CONTROLLED TEMPERATURE CHAIN

Traditionally vaccines are managed under strict temperature control (2°C–8°C) and within a system called the “cold chain” where vaccines are maintained at 2°C–8°C from the time vaccine is shipped by a manufacturer to the time the vaccine is given to a recipient. However, over the last several years it has become progressively clear that some vaccines, and particularly those that are lyophilized, can be quite thermostable. The PsA-TT vaccine is one such example. During the development of the vaccine, extensive stability studies were done at SIIL, and these studies showed that the vaccine was stable at elevated temperatures. Nonetheless, when the vaccine was licensed by the Drug Controller General of India (DCGI) in 2009, it was licensed for use at 2°C–8°C, like all other EPI antigens.

The thermal stability of PsA-TT was studied in more detail by SIIL with the help of PATH's Optimize Project and the regulatory help of Health Canada. This work resulted in a variation that was prepared and submitted to DCGI and later to WHO asking for permission to use the PsA-TT vaccine outside the cold chain. After detailed analysis of the stability data that was provided by SIIL, the DCGI granted permission that the PsA-TT conjugate vaccine could be safely exposed to temperatures as high as 40°C for 4 days without affecting the vaccine's ability to protect a vaccine recipient [16].

In collaboration with WHO, the government of Benin agreed to test the use of PsA-TT outside the cold chain under controlled conditions [16]. The vaccine could be taken outside the cold chain for as long as 4 days as long as the vaccine was not exposed to temperatures >40°C. Special vaccine vial monitors were made to change color at 40°C.

The obvious logistic advantages for such a change were that vaccines used outside the cold chain on the day of vaccination would no longer need ice packs; and that in remote and hard-to-reach areas, vaccination teams could be in the field, including overnight, for as long as 3 days without having to return each night to the health center for a fresh supply of ice packs.

For the controlled temperature chain study in Banikoara district (Benin) [16], a supply of PsA-TT vaccine was brought from national stores to the district level at 2°C–8°C. Special training was provided to health staff, and 155 000 persons were vaccinated outside the traditional 2°C–8°C cold chain [16]. The trial proceeded smoothly and the change did not result in any significant increase in vaccine wastage. There were no increases in AEFIs, and when vaccinators were interviewed after the trial, all agreed that the “controlled temperature chain” facilitated their work. When given a choice, all of the supervisors and almost all of the vaccinators (98%) said that they would prefer to use a controlled temperature chain in their next campaign. Last, a fiscal analysis revealed that not having to supply ice packs resulted in a decrease in logistic costs by 50% [16].

## CHALLENGES

Late release of operational funds and poor attendance of young males (16–29 years) at vaccination sessions were 2 problems that were repeatedly seen. The late release of operational funds in some countries negatively impacted the logistical management of the vaccine and the implementation of social mobilization activities. When funds reached the operational levels late, the critical microplanning exercises were also late or inappropriately conducted. As a result, the available resources were not appropriately allocated to meet the real operation needs, leading to various gaps in the implementation of the vaccination program. Among them, the most important problem was the late distribution of vaccines, logistics, and Information Education and Communication materials; the late development of social mobilization materials; the insufficient number of vaccination teams; a scarcity of team supervisors in the field; and an insufficient number of AEFI emergency kits. These important limitations were often overcome through intensive lobbying of government officials and an increase in the number of MVP country consultants during the planning of vaccination campaigns.

Almost everywhere, we observed a decreased attendance of young adult males (16–29 years) at vaccination posts. This situation was the most important challenge encountered during the implementation of the PsA-TT campaigns. Several reasons could explain the phenomenon, including that the long history of polio vaccination in some countries (eg, Nigeria) contributed to a belief in the communities that vaccines were only for children younger than 5 years. In other settings such as Burkina Faso, Mali, and Niger, other factors were contributory, including the seasonal migration of young men from Sahel countries to southern countries and sociocultural beliefs that men should not attend the same activities at the same time as women and children. Reinforcement of social mobilization activities, collaboration with influential groups in the communities, evening and farm vaccinations, and daily monitoring and feedback on vaccination team performance to supervisors helped vaccinators to reach more people. When all things were considered, the first 4 years of PsA-TT introduction were successful, despite some programmatic challenges, because of the flexibility and industry of campaign organization that was harmonized with social mobilization activities.

## SUMMARY

The first 5 years of PsA-TT introduction have been an unqualified success. The results that followed the initial introduction of PsA-TT in Burkina Faso were dramatic. The vaccine was safe, populations were desirous of the vaccine, and group A *N. meningitidis* meningitis rapidly disappeared after introduction. The results were positive and concrete and greatly facilitated the Gavi commitment to support PsA-TT introduction in the remaining meningitis belt countries. Each country introduction had its own set of problems, but these problems were as a rule surmounted. A cautionary note must be added. The initial strategy of achieving broad community protection has been successful, but the strategy will ultimately fail unless newborn cohorts and other at-risk groups, such as those born after completion of the initial campaigns, can be protected.
